# A combined gene expression and functional study reveals the crosstalk between N-Myc and differentiation-inducing microRNAs in neuroblastoma cells

**DOI:** 10.18632/oncotarget.12676

**Published:** 2016-10-15

**Authors:** Zhenze Zhao, Xiuye Ma, Spencer D. Shelton, Derek C. Sung, Monica Li, Daniel Hernandez, Maggie Zhang, Michael D. Losiewicz, Yidong Chen, Alexander Pertsemlidis, Xiaojie Yu, Yuanhang Liu, Liqin Du

**Affiliations:** ^1^ Department of Chemistry and Biochemistry at Texas State University, San Marcos, Texas, USA; ^2^ Greehey Children's Cancer Research Institute at UT Health Science Center at San Antonio, San Antonio, Texas, USA; ^3^ Division of Nutritional Sciences at Cornell University, Ithaca, New York, USA; ^4^ University of Texas at Austin, Austin, Texas, USA; ^5^ Department of Biology at Texas State University, San Marcos, Texas, USA; ^6^ Department of Biology, College of Sciences, University of Texas at San Antonio, San Antonio, Texas, USA; ^7^ Department of Chemistry & Biochemistry at St. Mary's University, San Antonio, Texas, USA; ^8^ Department of Epidemiology and Biostatistics, at UT Health Science Center at San Antonio, San Antonio, Texas, USA; ^9^ Department of Pediatrics, at UT Health Science Center at San Antonio, San Antonio, Texas, USA; ^10^ Cellular and Structural Biology, at UT Health Science Center at San Antonio, San Antonio, Texas, USA; ^11^ Graduate School of Biomedical Sciences at UT Health Science Center at San Antonio, San Antonio, Texas, USA

**Keywords:** neuroblastoma, microRNA, differentiation, MYCN

## Abstract

MYCN amplification is the most common genetic alteration in neuroblastoma and plays a critical role in neuroblastoma tumorigenesis. MYCN regulates neuroblastoma cell differentiation, which is one of the mechanisms underlying its oncogenic function. We recently identified a group of differentiation-inducing microRNAs. Given the demonstrated inter-regulation between MYCN and microRNAs, we speculated that MYCN and the differentiation-inducing microRNAs might form an interaction network to control the differentiation of neuroblastoma cells. In this study, we found that eight of the thirteen differentiation-inducing microRNAs, miR-506-3p, miR-124-3p, miR-449a, miR-34a-5p, miR-449b-5p, miR-103a-3p, miR-2110 and miR-34b-5p, inhibit N-Myc expression by either directly targeting the MYCN 3′UTR or through indirect regulations. Further investigation showed that both MYCN-dependent and MYCN-independent pathways play roles in mediating the differentiation-inducing function of miR-506-3p and miR-449a, two microRNAs that dramatically down-regulate MYCN expression. On the other hand, we found that N-Myc inhibits the expression of multiple differentiation-inducing microRNAs, suggesting that these miRNAs play a role in mediating the function of MYCN. In examining the published dataset collected from clinical neuroblastoma specimens, we found that expressions of two miRNAs, miR-137 and miR-2110, were significantly anti-correlated with MYCN mRNA levels, suggesting their interactions with MYCN play a clinically-relevant role in maintaining the MYCN and miRNA expression levels in neuroblastoma. Our findings altogether suggest that MYCN and differentiation-inducing miRNAs form an interaction network that play an important role in neuroblastoma tumorigenesis through regulating cell differentiation.

## INTRODUCTION

MYCN amplification occurs in about 20% of neuroblastoma cases and correlates with advanced-stage disease and poor patient outcomes [1–4]. *In vivo* studies have provided direct evidences demonstrating that MYCN overexpression is an important driving force of neuroblastoma development [5]. The MYCN-encoded protein N-Myc is a transcription factor that belongs to the Myc family of DNA binding basic region/helix-loop-helix/leucine zipper (bHLHZip) proteins [6]. Although its role in neuroblastoma tumorigenesis is not fully understood, studies have shown that N-Myc likely fulfills its oncogenic function through simultaneously stimulating expression of multiple oncogenic pathways and repressing expression of multiple tumor suppressive pathways [6, 7], and that inhibiting the differentiation of neuroblastoma cells is one of the important molecular mechanisms underlying its oncogenic function [8–10].

Recent studies suggest that microRNAs (miRNAs), a class of endogenously expressed, small non-coding RNAs that regulate gene expression at the translational level, play an important role in the MYCN-mediated oncogenic pathway [7]. On the one hand, MYCN has been demonstrated to regulate expression of many miRNAs in the context of several cancer types including neuroblastoma [11–13]. On the other hand, miRNAs have been indicated to regulate the expression of N-Myc levels at the translational level through directly targeting the 3′UTR of MYCN mRNA [7]. We recently identified a group of miRNAs that function as strong inducers of neuroblastoma cell differentiation [14]. Given the demonstrated inter-regulation between MYCN and microRNAs [7, 15–20], we speculate that MYCN and the differentiation-inducing miRNAs may form an interaction network that controls the differentiation process of neuroblastoma cells. In this study, we investigate whether the differentiation-inducing miRNAs regulated MYCN expression, whether N-Myc controls the expression of these miRNAs, and we further investigated whether N-Myc plays a role in mediating the differentiation-inducing functions of the miRNAs.

## RESULTS

### Differentiation-inducing miRNAs down-regulate MYCN expression at mRNA and protein levels

**Figure 1:**
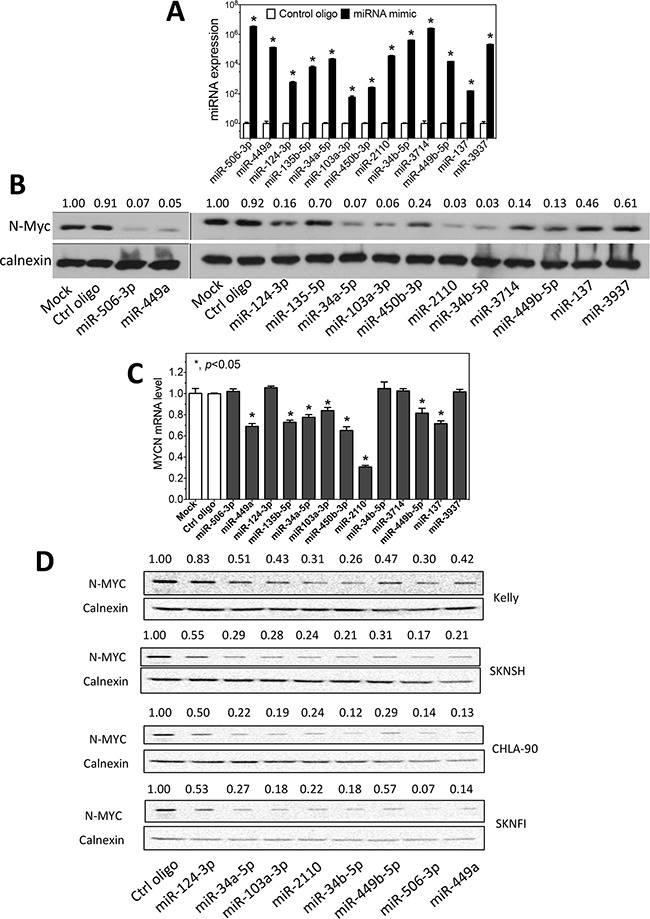
Regulation of N-myc expression by differentiation-inducing miRNAs **A.** Overexpression of miRNAs by the corresponding miRNA mimics in BE(2)-C cells. Cells were transfected with 25 nM mimics or control oligos. After two days, RNA was collected and miRNA levels were measured by qRT-PCR. Shown are the relative expression levels of miRNAs in cells transfected with the corresponding miRNA mimics normalized to those in cells transfected with control oligos. **B.** Effect of miRNA overexpression on N-Myc protein expression in BE(2)-C. N-Myc protein levels were measured by Western blots with calnexin as a loading control. Shown above the blots are the relative intensities of the corresponding bands. **C.** Effect of the miRNA overexpression on MYCN mRNA expression. MYCN expression was quantified using qRT-PCR and normalized with GAPDH mRNA as a loading control. Shown are fold changes of MYCN mRNA levels induced by miRNA mimics relative to mock transfection. *, *p*<0.05. **D.** Effect of miRNA overexpression on N-Myc protein expression in KELLY, SKNSH, CHLA-90 and SKNFI cells. N-Myc protein levels were measured as above.

In order to examine the role of differentiation-inducing miRNAs in regulating MYCN expression in neuroblastoma cells, we overexpressed a group of thirteen differentiation-inducing miRNAs that we identified previously [14] using miRNA mimics, synthetic oligonucleotides (oligos) used to raise intracellular miRNA levels, in a neuroblastoma cell line BE(2)-C, the cell line that we used to identify the differentiation-inducing miRNAs through high-content screening [14]. We then examined the effect of miRNA overexpression on the expression of MYCN at both mRNA and protein levels. The overexpression levels of the miRNAs by the corresponding miRNA mimics were confirmed by qRT-PCR, as shown in Figure 1A. As shown in Figure 1B, six of the thirteen miRNAs, which include miR-506-3p, miR-449a, miR-34a-5p, miR-103a-3p, miR-2110 and miR-34b-5p, dramatically down-regulated expression of MYCN at the protein level. Two miRNAs (miR-124-3p and miR-449b-5p) also down-regulate N-Myc protein expression but to a lesser extent. We further examined the effect of the thirteen miRNAs on MYCN expression at the mRNA level. As shown in Figure 1C, five miRNAs (miR-449a, miR-34a-5p, miR-103a-3p, miR-2110 and miR-449b-5p) that down-regulate N-Myc protein level also significantly down-regulated MYCN expression at the mRNA level. Interestingly, we found that three miRNAs (miR-506-3p, miR-124-3p and miR-34b-5p) that decreased N-Myc protein levels did not affect MYCN mRNA expression levels. On the other hand, two miRNA mimics (miR-135b-5p and miR-450b-3p) only significantly down-regulated MYCN mRNA expression; they did not dramatically affect the level of N-Myc protein expression.

For the eight miRNAs that down-regulated N-Myc protein expression in BE(2)-C cells, we further examined their effect on N-Myc expression in additional neuroblastoma cell lines with different genetic backgrounds, including MYCN-amplified and MYCN-nonamplified cell lines (Supplementary Table S1). As shown in Figure 1D, all eight miRNAs that down-regulated N-Myc protein expression to some extent in the four examined cell lines, although the extent of the N-Myc down-regulation by different miRNAs in different cell lines vary greatly.

Overall, these results suggest that multiple differentiation-inducing miRNAs play roles in regulating the expression of MYCN in neuroblastoma cells regardless of the genetic backgrounds of the cell lines.

### Differentiation-inducing miRNAs down-regulate MYCN expression through diverse molecular mechanisms

**Figure 2:**
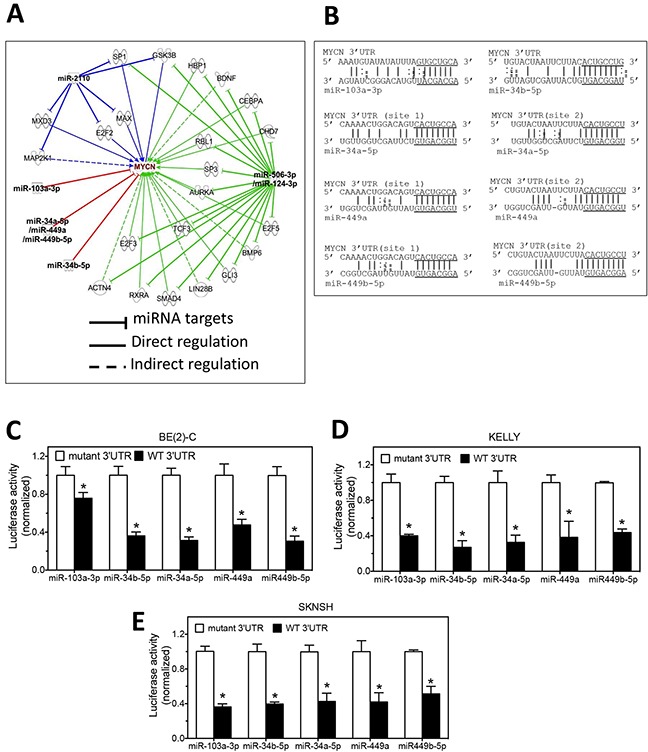
miRNAs regulate MYCN expression through direct and indirect pathways **A.** IPA analysis identifies the potential mechanisms by which the differentiation-inducing miRNAs regulate MYCN expression. **B.** The predicted interactions of miRNAs with the targets sites in the 3′UTR of MYCN mRNA. The seed sequences are underlined. **C-E.** Validation of the target sites of the miRNAs in the 3′UTRs of MYCN mRNA by luciferase reporter assay in BE(2)-C, KELLY and SKNSH cells. Cells were co-transfected with the luciferase reporter vector expressing either the wildtype 3′UTR of MYCN (WT 3′UTR) or mutated MYCN 3′UTR (mutant 3′UTR) and miRNA mimics. After 72 h of transfection, cells were lysed and luciferase activity was measured. Shown are normalized luciferase activities with the luciferase activity associated with the WT 3′UTR normalized to those associated with the corresponding mutant 3′UTRs. *, *p*<0.05 compared to the mutant 3′UTR.

The above investigation identified eight miRNAs that dramatically down-regulate MYCN expression at the protein level in neuroblastoma cells. We then exploited Ingenuity Pathway Analysis (IPA) (http://www.ingenuity.com) to identify potential molecular pathways by which these miRNAs may regulate MYCN expression. The IPA analysis identifies whether these miRNAs directly target the 3′UTR of MYCN mRNA, and also identifies the potential indirect signaling pathways by which the miRNAs may regulate MYCN expression. miRNA seed sequences have been demonstrated to play the dominant role in determining target specificity of miRNAs [21–23]. We therefore first grouped the eight miRNAs by their seed sequences in order to identify their predicted targets. As shown in Table 1, the eight miRNAs are grouped into two seed families and three miRNAs that have unique seed sequences. As shown in Figure 2A, the miR-34a-5p seed family (which includes miR-34a-5p, miR-449a and miR-449b-5p), miR-103a-3p and miR-34b-5p are predicted to directly target the 3′UTR of MYCN mRNA. Figure 2B shows the direct interactions of these miRNAs with their target sites in the MYCN mRNA 3′UTR. The MYCN mRNA 3′UTR contains two targets sites of the miR-34a-5p family. We further exploited the well-established luciferase report assay to validate the predicted targets sites [14, 24, 25]. As shown in Figure 2C, the miRNA over-expression significantly decreased luciferase activity in BE(2)-C cells expressing the wildtype MYCN 3′UTR comparing to the corresponding mutated 3′UTRs for all five miRNAs. The luciferase activities associated with the wildtype MYCN 3′UTR were decreased by >20% compared to the mutated 3′UTRs, consistent with what were observed in previous studies of miRNA targets using luciferase reporter vectors [26, 27]. Similar results were seen in KELLY and SKNSH cells, as shown in Figure 2D-2E.

**Table 1:** miRNA grouping based on seed sequences

Seed sequences (5′-3′)	miRNAs	Mature sequences 5′-3′
UAAGGCAC	hsa-miR-124-3p	UAAGGCACGCGGUGAAUGCC
	hsa-miR-506-3p	UAAGGCACCCUUCUGAGUAGA
	hsa-miR-449a	UGGCAGUGUAUUGUUAGCUGGU
UGGCAGUG	hsa-miR-34a-5p	UGGCAGUGUCUUAGCUGGUUGU
	hsa-miR-449b-5p	AGGCAGUGUAUUGUUAGCUGGC
UAGGCAGU	hsa-miR-34b-5p	UAGGCAGUGUCAUUAGCUGAUUG
AGCAGCAU	hsa-miR-103a-3p	AGCAGCAUUGUACAGGGCUAUGA
UUGGGGAA	hsa-miR-2110	UUGGGGAAACGGCCGCUGAGUG

Shown are the miRNA seed sequences, miRNAs that contain the seed sequence, and the mature sequences of the corresponding miRNAs.

The miR-506-3p seed family (which includes miR-506-3p and miR-124-3p) and miR-2110 do not directly target the MYCN 3′UTR. However, as shown in Figure 2A, these miRNAs are identified to potentially regulate MYCN expression through multiple signaling pathways. This potentially explains the significant down-regulation of N-Myc protein expression by these miRNAs.

### N-Myc regulates expression of differentiation-inducing miRNAs

**Figure 3:**
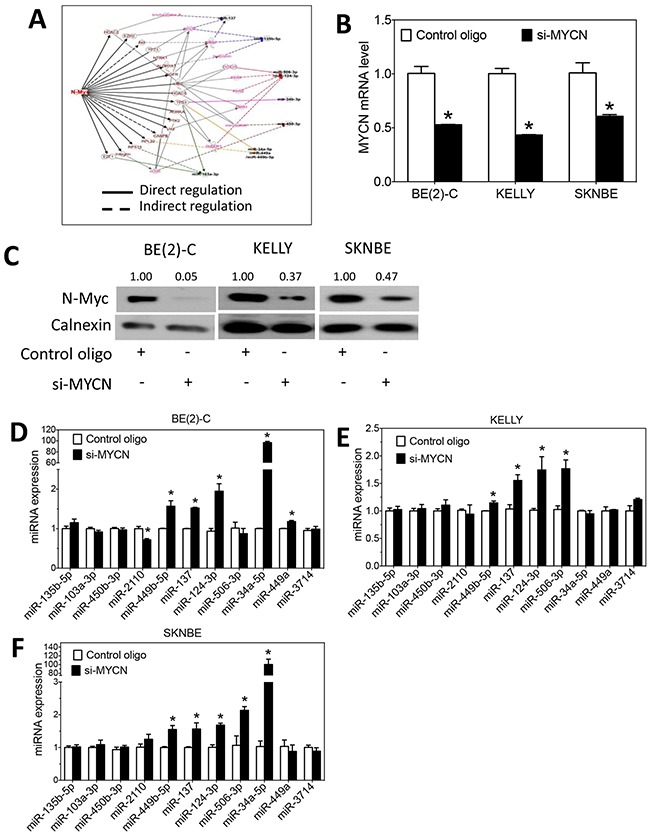
N-Myc regulates the expression of differentiation-inducing miRNAs **A.** IPA analysis identifies the potential mechanisms by whichN-Myc regulates the expression of the ten differentiation-inducing miRNAs. **B-C.** MYCN mRNA levels and N-Myc protein expression as a function of MYCN depletion. Cells were transfected with MYCN siRNA (si-MYCN) or control oligos for three days. (B) RNA was isolated for measuring MYCN mRNA levels as above, and (C) cell lysates were harvested for measuring N-Myc protein levels by Western blots as above. *, *p*<0.05 compared to control. **D-F.** Effect of MYCN knockdown on the expression of miRNAs. Cells were transfected with 25 nM of MYCN siRNA or control oligos. After 72 hours, RNA was collected, and miRNA levels were measured by qRT-PCR as above.

**Figure 4:**
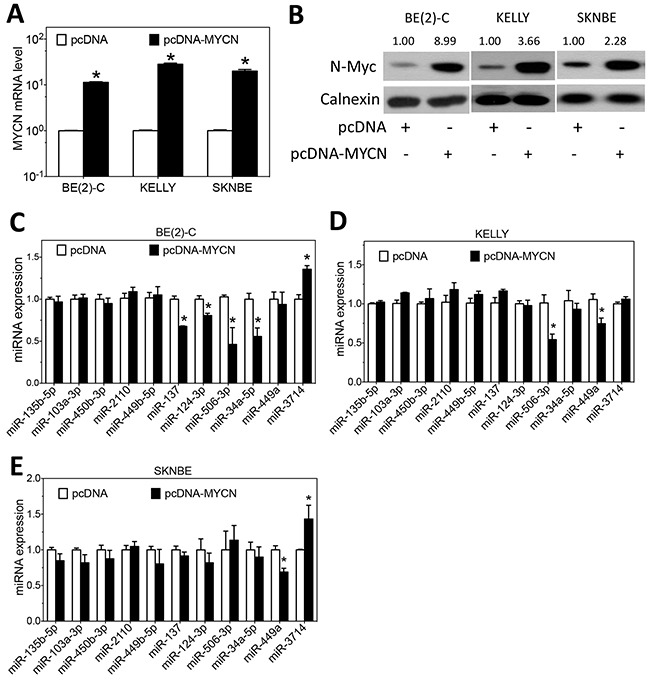
Effect of MYCN overexpression on the expression of differentiation-inducing miRNAs **A-B.** MYCN mRNA and N-Myc protein expression as a function of MYCN overexpression using the pcDNA-MYCN expression vector. Cells transfected with empty vector (pcDNA) were used as a negative control. Cells were transfected with the above vectors for three days, and (A) RNA was isolated for measuring MYCN mRNA levels as above, and (B) cell lysates were harvested for measuring N-Myc protein levels by Western blots as above. **C-E.** Effect of MYCN overexpression on the expression of miRNAs. Cells were transfected as above for 3 days, and miRNA levels were measured as above. (*, *p*<0.05, compared to control)

N-Myc is a transcription factor that regulates expression of many genes that control cell survival, growth and differentiation. The role of N-Myc in regulating miRNA expression has been reported previously [11–13]. We therefore comprehensively investigated whether expression of the thirteen differentiation-inducing miRNAs is regulated by N-Myc. We first used IPA to identify whether N-Myc is predicted to regulate expression of these miRNAs. As shown in Figure 3A, N-Myc is predicated to regulate ten of the thirteen differentiation-inducing miRNAs through multiple signaling pathways. Encouraged by these results, we examined whether MYCN knockdown affects the miRNA expression levels in neuroblastoma cells. The extent of MYCN mRNA and N-Myc protein depletion by the MYCN siRNA were confirmed by qRT-PCR and Western blots, respectively (Figure 3B–3C). Two of the thirteen miRNAs, miR-34b-5p and miR-3937 were not detectable or were only marginally detectable in the examined cell lines under any of the treatment conditions, so that the effect of MYCN overexpression or knockdown on their expression could not be reliably evaluated. For the rest eleven miRNAs, as shown in Figure 3D-3F, siRNA knockdown of MYCN expression leads to a significant upregulation of six miRNAs (miR-449b-5p, miR-137, miR-124-3p, miR-34a-5p, miR-449a and miR-506-3p) in at least one of the three neuroblastoma cell lines examined, indicating that N-Myc functions as a repressor of these miRNA expressions. One exception is miR-2110, which is observed to be slightly but significantly down-regulated upon MYCN knockdown in BE(2)-C cells, as shown in Figure 3D. We further examined the effect of MYCN overexpression on miRNA expression. The overexpression of MYCN at the mRNA and protein levels by pcDNA-MYCN expression vector were confirmed, as shown in Figure 4A-4B. As shown in Figure 4C-4E, overexpression of MYCN significantly down-regulated the expression of five miRNAs (miR-137, miR-124-3p, miR-34a-5p, miR-449a and miR-506-3p) in at least one of the three cell lines, which is consistent with their up-regulation by MYCN knockdown. Contrary to the majority of the miRNAs whose expression is suppressed by MYCN overexpression, miR-3714 expression was significantly upregulated by MYCN overexpression in BE(2)-C and SKNBE cells.

Overall, the results show that MYCN mainly functions as a repressor of the expression of differentiation-inducing miRNAs in neuroblastoma cells by negatively regulating the expression of six differentiation-inducing miRNAs (miR-449b-5p, miR-137, miR-124-3p, miR-34a-5p, miR-449a and miR-506-3p) in at least one of the three cell lines. However, a minor stream of positive regulation by MYCN is observed for two miRNAs (miR-2110 and miR-3714) in certain cell lines.

### MYCN plays a role in mediating the differentiation-inducing function of miR-506-3p and miR-449a

**Figure 5:**
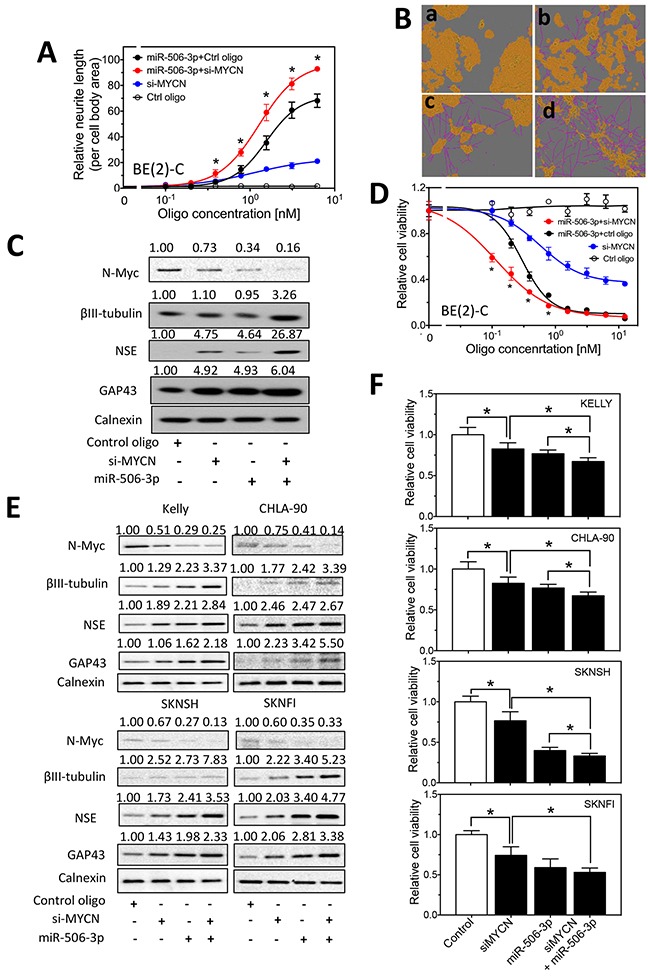
Effect of MYCN knockdown and miR-506-3p overexpression on cell differentiation and cell survival in neuroblastoma cells **A-B.** Effect of MYCN knockdown and miR-506-3p overexpression on neurite outgrowth in BE(2)-C cells. Cells were transfected with different concentrations of the indicated oligos. After 4 days, relative neurite outgrowth was measured. (A) Quantification of neurites showing the dose-dependent effect of MYCN siRNA (si-MYCN) and miR-506-3p mimic on neurite outgrowth. (B) Representative images, with the cell body area (yellow) and neurites (pink) defined, showing the effect of si-MYCN and miR-506-3p mimic on neurite outgrowth. Shown are cell images for **(a)** control oligo (6 nM), **(b)** si-MYCN (3 nM), **(c)** miR-506-3p mimic (3 nM) and **(d)** si-MYCN (3 nM)+miR-506-3p mimic (3 nM). **C.** Effect of MYCN knockdown and miR-506-3p overexpression on the expression of *N-Myc and* differentiation markers in BE(2)-C cells. Cells were transfected with the indicated oligos. After 4 days, cell lysates were collected, and protein levels of N-Myc, as well as protein levels of the differentiation markers, βIII–tubulin, NSE and GAP43 were measured by Western blots with calnexin levels as a loading control. **D.** Effect of MYCN knockdown and miR-506-3p overexpression on cell viability of BE(2)-C cells. Cells were transfected with different concentrations of the indicated oligos. After 4 days, cell viability were measured as described in the Material and Methods. *, p<0.05 compared cells co-transfected with si-MYCN and miR-506-3p mimic to cells co-transfected with control oligo and miR-506-3p mimic. **E.** Effect of MYCN knockdown and miR-506-3p overexpression on expression of N-Myc and differentiation markers in KELLY, CHLA-90, SKNSH and SKNFI cells. **F.** Effect of MYCN knockdown and miR-506-3p overexpression on cell viability in KELLY, CHLA-90, SKNSH and SKNFI cells. *, *p*<0.05.

**Figure 6:**
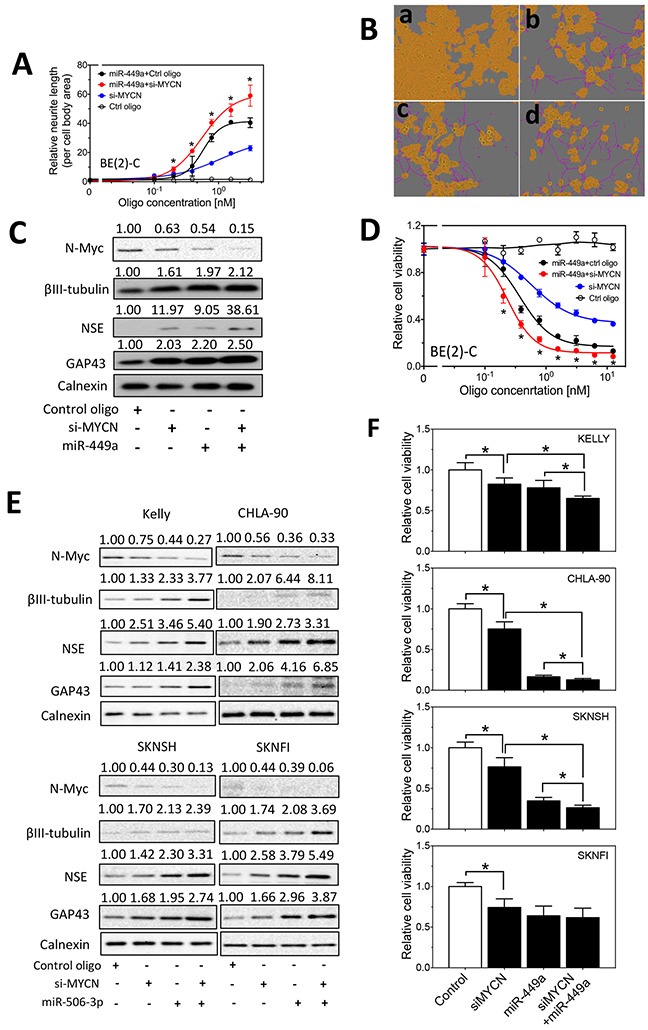
Effect of MYCN knockdown and miR-449a overexpression on cell differentiation and cell survival in neuroblastoma cells **A-D.** Effect of MYCN knockdown and miR-449a overexpression on neurite outgrowth (A-B), on expression of *N-Myc* and differentiation markers (C), and on cell viability (D) in BE(2)-C cells as measured as above. Shown in (B) are cell images for (**a**) control oligo (6 nM), (**b**) si-MYCN (3 nM), (**c**) miR-449a mimic (3 nM) and (**d**) si-MYCN (3 nM)+miR-449a mimic (3 nM). *, p<0.05 compared cells co-transfected with si-MYCN and miR-506-3p mimic to cells co-transfected with control oligo and miR-506-3p mimic. **E.** Effect of MYCN knockdown and miR-449a overexpression on expression of N-Myc and differentiation markers in KELLY, CHLA-90, SKNSH and SKNFI cells. **F.** Effect of MYCN knockdown and miR-449a overexpression on cell viability in KELLY, CHLA-90, SKNSH and SKNFI cells. *, *p*<0.05.

In order to understand the role of MYCN in mediating the function of the differentiation-inducing miRNAs, we first examined whether knocking down MYCN expression potentiates the differentiation-inducing effect of miR-506-3p and miR-449a, two miRNAs that dramatically down-regulate N-Myc protein expression level, in BE(2)-C cells. As shown in Figure 5A-B, MYCN knockdown or miR-506-3p overexpression alone stimulates neurite outgrowth, and combined miR-506-3p overexpression and MYCN knockdown lead to significantly enhanced neurite outgrowth. The extent of N-Myc protein down-regulation by siMYCN and miR-506-3p mimic was confirmed (Figure 5C). Figure 5C further shows that combined miR-506-3p overexpression and MYCN knockdown lead to enhanced expression of the neuroblastoma differentiation markers βIII–tubulin, neuron specific enolase (NSE) and growth associated protein 43 (GAP43) [28–34], indicating that cell differentiation is truly induced. Correspondingly, as shown in Figure 5D, combined miR-506-3p overexpression and MYCN knockdown also lead to enhanced reduction of cell viability, indicating that reduced cell growth/survival is coupled with the enhanced cell differentiation. We further examined combined MYCN knockdown and miR-506-3p overexpression in additional neuroblastoma cell lines. As shown in Figure 5E–5F, combined siMYCN and miR-506-3p mimic treatment shows enhanced expression of differentiation markers and reduced cell viability comparing to either MYCN knockdown or miR-506-3p in all the four cell lines, although the decrease of cell viability by combined siMYCN and miR-506-3p mimic treatment relative to miR-506-3p treatment alone did not reach statistical significance in SKNFI cells. Similar results were observed for the combined MYCN knockdown and miR-449a overexpression in the five cells lines, as shown in Figure 6A-6F. Overall, the results suggest that the differentiation-inducing functions of miR-506-3P and miR-449a are at least partially, if not completely, mediated by down-regulating MYCN expression in neuroblastoma cell lines.

**Figure 7:**
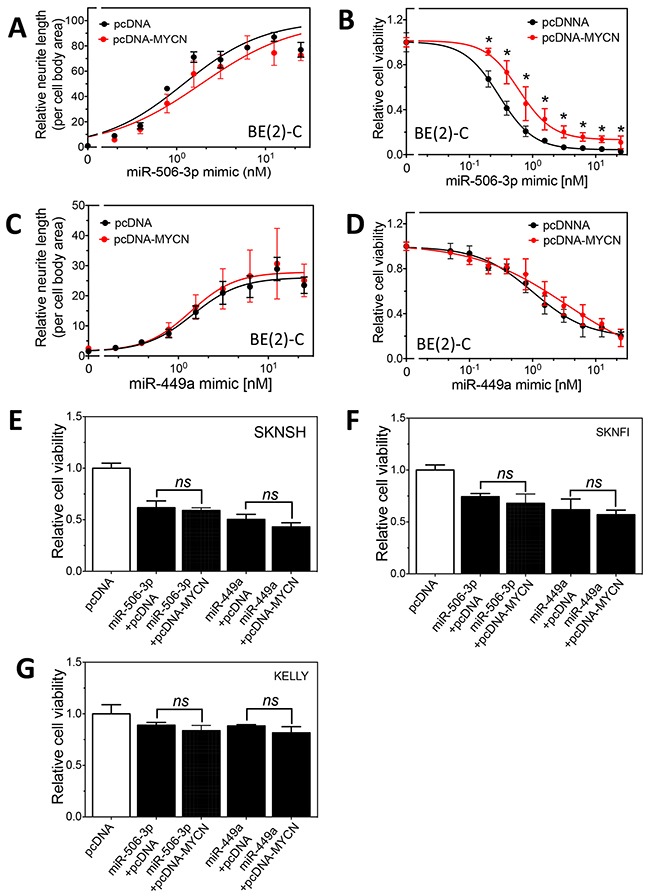
Effect of MYCN overexpression on the differentiation-inducing effect of miR-506-3p and miR-449a in neuroblastoma cells **A-B.** Effect of MYCN overexpression on miR-506-3p mimic-induced cell differentiation and cell survival reduction in BE(2)-C cells. Cells expressing pcDNA-MYCN or pcDNA were transfected with different concentrations of miR-506-3p mimic. After 4 days, neurite outgrowth (A) and cell viability (B) were measured as above. **C-D.** Effect of MYCN overexpression on miR-449a mimic-induced cell differentiation and cell survival reduction in BE(2)-C cells. Cells were transfected with different concentrations of miR-449a mimic, and neurite outgrowth (C) and cell viability (D) were measured as above. **E-G.** Effect of MYCN overexpression on miR-506-3p mimic- and miR-449a-induced cell survival reduction in SKNSH, SKNFI and Kelly cells. *, p<0.05 compared to the control pcDNA.

We next examined whether MYCN overexpression blocks the differentiation-inducing effect of miR-506-3p and miR-449a in BE(2)-C cells. As shown in Figure 7A, MYCN overexpression only slightly, but not significantly inhibit the effect of miR-506-3p on neurite outgrowth in BE(2)-C cells. Correspondingly, as shown in Figure 7B, MYCN overexpression inhibits the effect of miR-506-3p on reducing cell survival to a very minor extent. For miR-449a, MYCN overexpression does not show detectable effect on miR-449a mimic-induced neurite outgrowth and cell survival inhibition, as shown in Figure 7C-7D. Similarly, MYCN overexpression in SKNSH, and SKNFI and KELLY did not significantly inhibit the effect of miR-506-3p and miR-449a mimic on cell survival, as shown in Figure 7E-7G. Together, these results indicate that MYCN overexpression does not sufficiently block the differentiation-inducing effect of miR-506-3p and miR-449a, and that the two miRNAs can overcome the MYCN oncogenic pathway and exert strong differentiation-inducing effects in neuroblastoma cells.

We further examined whether anti-miR-506-3p and anti-miR-449a inhibitors affect N-Myc expression and siMYCN-induced cell differentiation. We found that anti-miRs did not affect N-Myc expression, and that anti-miRs did not significantly affect the effect of siMYCN on cell survival (Supplementary Figure S1).

### Expression levels of miR-2110 and miR-137 are significantly anti-correlated with MYCN mRNA levels in neuroblastoma tumor specimens

In order to examine the clinical relevance of the crosstalk between the differentiation-inducing miRNAs and MYCN in neuroblastoma, we analyzed the correlations of the miRNA expression with MYCN mRNA expression levels in neuroblastoma specimens from SEQC-seqcnb1 dataset. As shown in Table 2, expressions of two miRNAs, miR-2110 and miR-137, are significantly anti-correlated with MYCN mRNA levels. Three miRNAs, miR-506-3p, miR-449a and miR-449b-5p, are not detectable in the examined tumor tissues. The rest eight miRNAs are not significantly correlated with MYCN expressions.

**Table 2:** Correlations of miRNA with MYCN mRNA levels

miRNA name	*r*	*p*
miR-124-3p	0.101	0.02
miR-135b-5p	−0.08	0.07
miR-506-3p	Not detectable	
miR-34a-5p	−0.019	0.66
miR-103a-3p	0.048	0.28
miR-450b-3p	0.011	0.81
miR-449a	Not detectable	
miR-2110	−0.235	1.20E-07*
miR-34b-5p	0.062	0.17
miR-107	−0.044	0.33
miR-3714	−0.062	0.17
miR-449b-5p	Not detectable	
miR-137	−0.274	5.20E-10*
miR-3937	0.107	0.02

Shown are the miRNA names, *r* values and *p* values of the correlations. The correlations were assessed by Pearson correlation.

* , *p*<0.01 considered statistically significant.

## DISCUSSION

In this study, we investigated the role of a group of differentiation-inducing miRNAs in regulating MYCN expression. Our results show that multiple differentiation-inducing miRNAs inhibit MYCN expression, among which miR-34a and miR-449a have been previously identified to down-regulate MYCN expression by directly targeting the 3′UTR of MYCN mRNA [7, 15–18]. In addition to recapitulating the previous findings, our study identified additional miRNAs, which include miR-506-3p, miR-103a-3p, miR-2110, miR-34b-5p, miR-124-3p and miR-449b-5p, that inhibit MYCN expression through either direct or indirect regulatory mechanisms. These findings expand the family of miRNAs that participate in the signaling network that modulates MYCN expression in neuroblastoma cells.

In investigating the role of MYCN in mediating the miRNA differentiation-inducing function, we found that MYCN knockdown significantly induces cell differentiation, which is consistent with previous findings [8]. Furthermore, we found that combining MYCN knockdown with overexpression of either miR-506-3p or miR-449a leads to significantly enhanced neuroblastoma cell differentiation comparing to miRNA overexpression alone. Coupled with the dramatic down-regulation of N-Myc expression by miR-506-3p and miR-449a overexpression, our results suggest that at least one of the mechanisms by which miR-506-3p and miR-449a induce cell differentiation is through down-regulating MYCN expression. Unexpectedly but interestingly, despite the observed significant effect of MYCN knockdown on inducing neuroblastoma cell differentiation, we found that MYCN overexpression does not have a dramatic effect on inhibiting the differentiation-inducing function of miR-506-3p and miR-449a. These results suggest that miR-506-3p and miR-449a induce neuroblastoma cell differentiation through multiple molecular pathways, and that MYCN only plays a partial role in mediating the miRNA differentiation-inducing function. When the MYCN-mediated oncogenic pathway is overexpressed, the miRNAs are able to bypass this pathway and exert strong differentiation-inducing effects through activation of MYCN-independent cell differentiation pathways. In addition, we found that overexpression of several differentiation-inducing miRNAs does not affect the expression level of MYCN, suggesting that their differentiation-inducing function is entirely independent of MYCN-mediated signaling pathways. The independence of the miRNA differentiation-inducing functions from the oncogenic function of MYCN supports the general therapeutic potential of these miRNAs — the miRNAs induce cell differentiation regardless of MYCN expression status in the neuroblastoma cells, and they therefore can be potentially used to treat both MYCN-amplified and MYCN-nonamplified neuroblastomas.

In this study, we also characterized the role of MYCN in regulating expression of the differentiation-inducing miRNAs, and we found that N-Myc functions as a negative regulator of expression of multiple differentiation-inducing miRNAs. This investigation also recapitulates a previous finding — miR-137 has been previously shown to be down-regulated by MYCN in neuroblastoma [19, 20]. Our further analysis of the neuroblastoma specimens shows that expression of miR-137 was significantly anti-correlated with MYCN mRNA expression in neuroblastoma specimens. Altogether, this evidence supports the strong function of MYCN in controlling miR-137 expression in neuroblastoma. Besides miR-137, we identified for the first time that MYCN negatively regulates the expression of miR-506-3p, miR-449b-5p, miR-124-3p and miR-34a-5p, which expands the spectrum of miRNAs regulated by MYCN. Although MYCN has been demonstrated to regulate neuroblastoma cell differentiation, the mechanisms underlying such function are not clearly defined. The fact that MYCN represses the expression of multiple differentiation-inducing miRNAs suggests that one of the key mechanisms by which MYCN inhibits neuroblastoma cell differentiation is through simultaneously repressing expression of a group of miRNAs that function as inducers of neuroblastoma cell differentiation.

Contrary to the down-regulation of expressions by MYCN for the majority of the differentiation-inducing miRNAs, we found that MYCN overexpression up-regulates the expression of miR-3714 in BE(2)-C and SKNBE cells. The up-regulation of a differentiation-inducing miRNA induced by MYCN could be an important self-defense mechanism against the oncogenic signaling pathways during neuroblastoma tumorigenesis. In other words, in response to the pathologically elevated MYCN oncogenic pathway, the neuroblastoma cells activates the differentiation-inducing miRNA-mediated tumor suppressive pathway in order to prevent oncogenic transformation of the cells. The significance of the activated tumor suppressive mechanism by MYCN certainly warrants further investigation in future studies — elucidating the self-defensive tumor suppressive mechanisms during neuroblastoma tumorigenesis may reveal novel therapeutic targets for treating neuroblastoma. On the other hand, miR-2110 is down-regulated upon MYCN knockdown in KELLY cells. The down-regulation of a differentiation-inducing miRNA upon MYCN knockdown could be an important mechanism that causes the resistance to anti-cancer drugs targeting the MYCN-mediated oncogenic pathway — the significance of this putative anti-cancer resistant mechanism certainly should be further evaluated in future studies.

In examining the expression levels of the 13 differentiation-inducing miRNAs and MYCN mRNA in neuroblastoma specimens, we found that two miRNAs, miR-137 and miR-2110, are significantly anti-correlated with MYCN mRNA levels. The negative correlation of miR-137 with MYCN is likely due to the repression of miR-137 expression by MYCN as discussed above, since there is no current evidence supporting the regulation of MYCN by miR-137. On the contrary, according to current findings, the negative correlation between miR-2110 and MYCN expressions is likely due to the repression of MYCN expression by miR-2110. We are first to discover the inhibition of MYCN expression by miR-2110. Although our results show that MYCN overexpression slightly up-regulates miR-2110 in one of the investigated cell lines, the significant negative correlation between MYCN mRNA and miR-2110 levels in neuroblastoma specimens suggest that the regulation of MYCN expression by miR-2110 is the main stream in the interaction between MYCN and miR-2110, whereas the regulation of miR-2110 by MYCN does not play a significant role in determining the correlation between their expressions. Studies are certainly needed to further address the interactions between miR-2110 and MYCN in larger clinical and experimental settings and to define the mechanisms underlying their interactions. Eight of the thirteen differentiation-inducing miRNAs are not significantly correlated with MYCN mRNA expressions. This, however, does not necessarily mean that these miRNAs are not important in controlling MYCN expression. Since multiple miRNAs have the function of regulating MYCN expression, it is reasonable to speculate that it is their concerted activities that make a significant contribution to control the MYCN expression. How to evaluate the concerted activities of these miRNAs in clinical settings warrants further investigation in the future.

Interestingly, three miRNAs, miR-506-3p, miR-449b-5p and miR-449a, are not detectable in neuroblastoma specimens. This observation is particularly interesting since it suggests that the lack of expression of these differentiation-inducing miRNAs may be a key factor for blocking the cell differentiation pathway during neuroblastoma tumorigenesis. Consistent with the observed low expressions in neuroblastoma specimens, we found that their expressions in neuroblastoma cell lines are also low, and can only be detected with large RNA sample input. As expected, when using anti-miR-506-3p and anti-miR-449a inhibitors to deplete their expressions in neuroblastoma cells, we were unable to observe a significant effect of the anti-miRs on N-Myc expression and cell differentiation. This can be explained by the low expression of these two miRNAs in neuroblastoma cells. Since their expression levels are so low, further depletion of their expressions in neuroblastoma cells would not have a dramatic effect on its target gene (MYCN) expression. This further explains why replacement of differentiation-inducing miRNAs in neuroblastoma cells has such a dramatic differentiation-inducing effect.

The mechanisms by which the differentiation-inducing miRNAs regulate MYCN expression as well as the mechanisms by which MYCN regulates the expression of these miRNAs need to be further investigated. In this study, we only characterized the regulation of MYCN expression as a direct target of the differentiation-inducing miRNAs. Several miRNAs, including miR-506-3p and miR-2110, are strong repressors of MYCN expression but do not directly target the 3′UTR of MYCN mRNA. Our informatics analysis indicates that each of these miRNAs potentially regulate MYCN expression through multiple signaling pathways. Each of these pathways needs to be investigated individually in order to identify which pathway(s) plays a key role in mediating the regulation of MYCN by these miRNAs. Likewise, the identified potential signaling pathways by which MYCN regulates the miRNA expression also need to be further characterized in future studies.

In summary, our results indicate that MYCN and differentiation-inducing miRNAs form a complicated interaction network in neuroblastoma cells, and we identified novel miRNA:MYCN inter-regulations that have not been identified previously. Given the demonstrated critical role of both MYCN and the differentiation-inducing miRNAs in regulating neuroblastoma cell differentiation, our findings suggest that the imbalance of this interaction network — caused by either overexpression of MYCN or repression of miRNA expression — may be an important driving force of neuroblastoma tumorigenesis. In the future, comparing the expressions of these miRNAs in neuroblastoma specimens to that in normal adjacent tissues will be the first step to understand whether loss of the expression of the differentiation-inducing miRNAs is one of the mechanisms contributing to neuroblastoma tumorigenesis. The molecular mechanisms underlying the inter-regulation between MYCN and the differentiation-inducing microRNAs also need to be further elucidated. In addition, our study suggests that down-regulating MYCN only plays a partial role in mediating the differentiation-inducing functions of the microRNAs. The MYCN-independent mechanisms underlying their differentiation-inducing function certainly warrant further investigation.

## MATERIALS AND METHODS

### Materials

miRNA mimics and negative control oligo were purchased from Dharmacon. Anti-miR-506-3p and anti-miR-449a were from Ambion, Inc. siRNAs against MYCN were purchased from Sigma. Rabbit anti-MYCN was purchased from Cell Signaling. Rabbit anti-GAP43, anti-NSE and anti-βIII-tubulin antibodies were obtained from Abcam. Rabbit anti-calnexin antibody, and goat anti-rabbit secondary antibody conjugated with horseradish peroxidase (HRP) were from Santa Cruz (Dallas, TX, USA).

### Cell lines

BE(2)-C, SKNBE and SKNSH cells were purchased from the American Type Culture Collection (ATCC). BE(2)-C is a clonal subline of the SKNBE. KELLY cells were obtained from the cell line repository at the Greehey Children's Cancer Research Institute, University of Texas Health Science Center at San Antonio. CHLA-90 cells were obtained from Children's Oncology Group. Cells were grown in DMEM/F12 supplemented with 10% fetal bovine serum.

### Detection and quantification of neurite outgrowth

Neurite outgrowth was quantified as described previously [14]. Briefly, cells were plated and treated in 96-well plates. After 4 days, cells were placed into the ZOOM IncuCyte Imaging System (Essen BioScience), and images were taken under 20X magnification. The relative neurite length was then calculated using the neurite definition determined for each specific cell line using the NeuroTrack system.

### Luciferase reporter assay

The wildtype 3′UTR of MYCN containing the predicted target sites of the miRNAs were cloned from human genomic DNA. Mutant constructs with the seed target sequences for the corresponding miRNAs deleted were generated by site-directed mutagenesis. The 3′UTRs were cloned downstream of the firefly luciferase coding sequences into the pmirGLO dual-luciferase reporter (Promega) as previously described [14]. BE(2)-C cells were co-transfected with 0.8 ng/ul of the luciferase reporters and 25 nM of the mimics in 96-well plates. After 72 hours, Firefly and Renilla luciferase activities were measured using the Dual-Glo Luciferase Assay System (Promega). Firefly luciferase activity was normalized to Renilla luciferase activity to evaluate the regulation of firefly luciferase expression by the miRNAs.

### Western blots

Cells were harvested and cell lysates were prepared using RIPA buffer. Protein concentration was determined using the Pierce BCA assay (Thermo Fisher Scientific). Equal amounts of cell lysates were resolved by SDS-PAGE, and proteins were transferred to the PVDF membranes (Bio-Rad Laboratories). The membranes were blocked and probed with specific primary antibodies; proteins were then detected using the corresponding secondary antibodies and visualized by enhanced chemiluminescent (ECL) substrate (Pierce). The intensities of the immune-bands were quantified using ImageJ software.

### Cell viability assay

Cells were plated and were transfected in 96-well plates. After 4 days of culture, cell viability was determined using the CellTiter-Glo Luminescent Cell Viability Assay (Promega).

### Construction of the MYCN expression vector

The protein-coding region of the MYCN gene was amplified by PCR from a human MYCN cDNA clone (Origene, SC116780). The amplified MYCN sequence was inserted into the HindIII and EcoRI restriction sites of the multiple cloning site of expression vector pcDNA3.1+ to generate MYCN expression vector pcDNA-MYCN. The inserted sequences were verified by sequencing.

### mRNA and miRNA expression

Total RNA was isolated using the mirVana RNA isolation kit (Life Technologies) as previously described [35]. miRNA expression was measured by qRT-PCR using TaqMan microRNA Assays (Life Technologies) with expression of RNU44 RNA used as a loading control. MYCN mRNA expression was measured by qRT-PCR using TaqMan gene Assays (Life Technologies) with expression of GAPDH mRNA used as a loading control.

### Correlation of MYCN mRNA with miRNA levels in neuroblastoma specimens

The correlation analyses were based on the neuroblastoma SEQC-Seqcnb1 dataset collected from 498 neuroblastoma specimens and published in the R2: Genomics Analysis and Visualization Platform (http://r2.amc.nl). The mRNA and miRNA expression data in this dataset were generated using the Illumina HiSeq 2000 platform [36]. The correlations of the MYCN mRNA with miRNA levels were analyzed using Pearson correlation, with *p* < 0.01 considered statistically significant.

### Statistical analysis

The statistical significance for each treatment was determined by t-test by comparing the treatment group with control, with p< 0.05 considered statistically significant.

## SUPPLEMENTARY FIGURE AND TABLE


