# Supplementary material for: A combined gene expression and functional study reveals the crosstalk between N-Myc and differentiation-inducing microRNAs in neuroblastoma cells
Source: Oncotarget. 2016 Oct 15;7(48):79372–87. doi: 10.18632/oncotarget.12676 (PMC5346721; doi:10.18632/oncotarget.12676)
Supplement: Supplementary file 1 [file oncotarget-07-79372-s001.pdf]

A combined gene expression and functional study reveals the crosstalk between N-Myc and differentiation-inducing microRNAs in neuroblastoma cells

SUPPLEMENTARY FIGURE AND TABLE

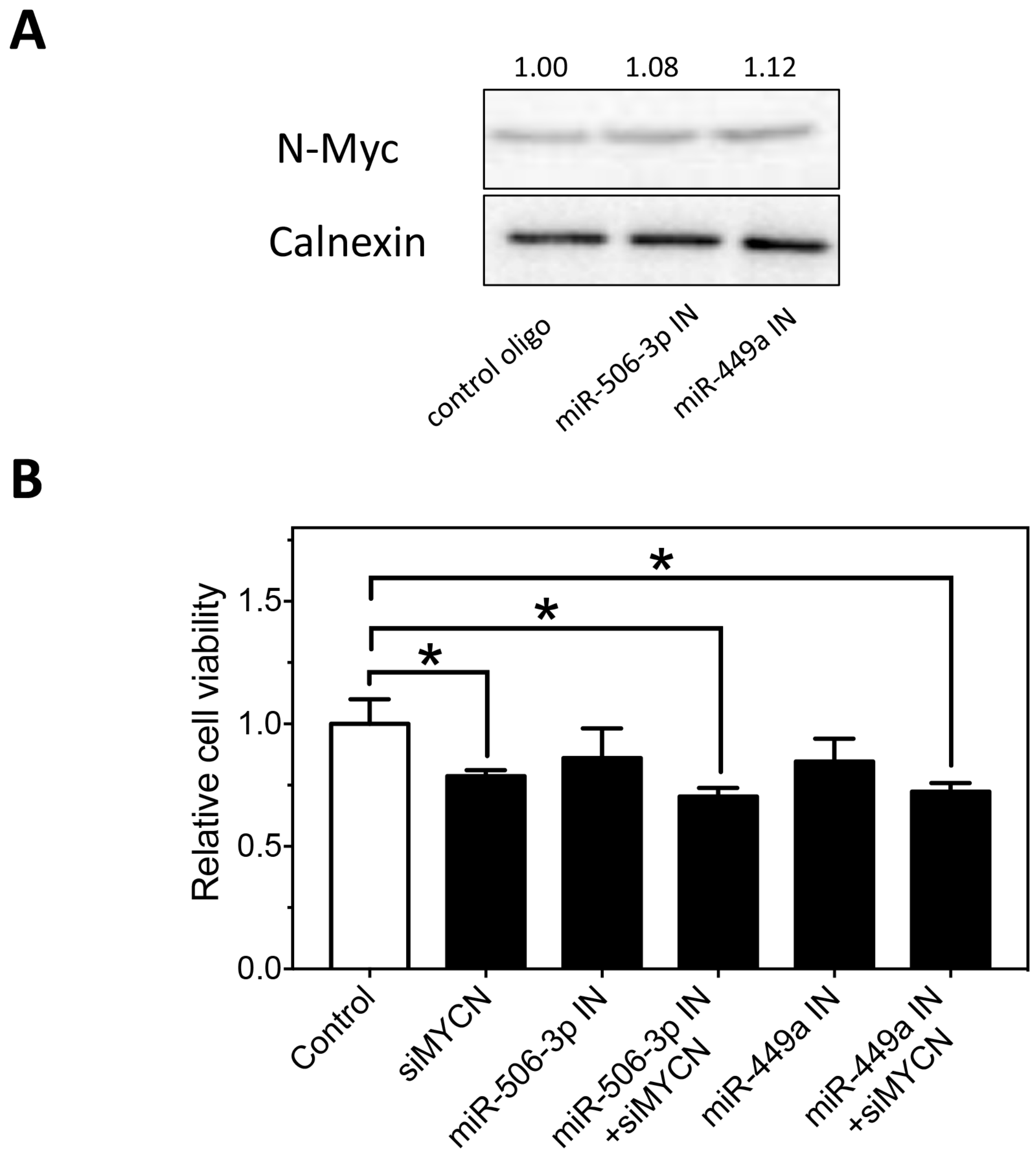

Supplementary Figure S1: Effect of anti-miRNAs on N-Myc expression and on cell differentiation. The indicated neuroblastoma cells were transfected with 25nM anti-miR-506-3p (miR-506-3p IN), anti-miR-449a (miR-449a IN), control oligos and / or siMYCN for 4 days. **A.** Protein lysates were collected and N-Myc protein levels were measured by Western blots as described in Figure 1 legend, with calnexin as a loading control. **B-D.** Cell viability was measured as described in Figure 5 legend. *, $p<0.05$ compared to the control.

Supplementary Table S1: Genetic backgrounds of neuroblastoma cell lines used in the study

Cell line	Age (yr)	Gender	Stage	Chr 1p alteration	Chr 17 alteration	MYCN ampl.
BE(2)-C	2	male	4	Y	N	Y
SKNBE	1.8	male	4	Y	N	Y
SKNSH	4	female	4	N	N	N
CHLA-90	8.5	male	4	unk	unk	N
SKNFI	11	male	unk	unk	unk	N
KELLY	unk	unk	unk	unk	unk	Y

Shown are the name of the cell line, age and gender of the patient, stage of the tumor from which the cell lines are derived, chromosome 1p and 17 alterations, and MYCN gene amplification status. *unk, unknown; Chr, Chromosome; ampl, amplification.
